# A novel data-driven methodology for influenza outbreak detection and prediction

**DOI:** 10.1038/s41598-021-92484-6

**Published:** 2021-06-24

**Authors:** Lin Du, Yan Pang

**Affiliations:** 1 Business Analytics Centre, National University of Singapore, Singapore, 119613 Singapore; 2 Department of Analytics and Operations, National University of Singapore, Singapore, 119613 Singapore; 3 Data Analytics, Zuellig Pharma Holdings Pte. Ltd., Singapore, 228233 Singapore

**Keywords:** Health services, Public health

## Abstract

Influenza is an infectious disease that leads to an estimated 5 million cases of severe illness and 650,000 respiratory deaths worldwide each year. The early detection and prediction of influenza outbreaks are crucial for efficient resource planning to save patient’s lives and healthcare costs. We propose a new data-driven methodology for influenza outbreak detection and prediction at very local levels. A doctor’s diagnostic dataset of influenza-like illness from more than 3000 clinics in Malaysia is used in this study because these diagnostic data are reliable and can be captured promptly. A new region index (RI) of the influenza outbreak is proposed based on the diagnostic dataset. By analysing the anomalies in the weekly RI value, potential outbreaks are identified using statistical methods. An ensemble learning method is developed to predict potential influenza outbreaks. Cross-validation is conducted to optimize the hyperparameters of the ensemble model. A testing data set is used to provide an unbiased evaluation of the model. The proposed methodology is shown to be sensitive and accurate at influenza outbreak prediction, with average of 75% recall, 74% precision, and 83% accuracy scores across five regions in Malaysia. The results are also validated by Google Flu Trends data, news reports, and surveillance data released by World Health Organization.

## Introduction

The World Health Organization (WHO) released the top 10 issues that required attention in 2019, of which infectious diseases dominated the list. Influenza is one of them^1^. Influenza is a highly contagious respiratory tract infection that causes diseases ranging from mild respiratory tract infection (RTI) to severe pneumonia and even death. Worldwide, seasonal influenza leads to an estimated 5 million cases of severe illness and 650,000 respiratory deaths every year^2^. It also causes a significant hospitalization burden, workplace absences, and productivity losses. For example, based on a study conducted at the University of Malaya Medical Centre in Malaysia in 2009, the direct healthcare cost for each hospitalized H1N1 patient was USD 510, which was 60% higher than the year 2007 per capita national expenditure on health of USD 318^3^. Given these circumstances, investigators are working on detecting and predicting influenza outbreaks early. This prediction would bring tremendous value to the world’s healthcare systems. Firstly, the early detection of influenza outbreaks is crucial to the healthcare system to enable efficient resource planning and save healthcare costs. Secondly, early detection can potentially help save people’s lives. Thirdly, we can control the spread of influenza if we can predict it early.

Traditional surveillance is widely used to monitor anomalies in influenza-like illness (ILI) cases in selected hospitals or clinics. For example, in Malaysia, the Ministry of Health designs and deploys a system to monitor national influenza status efficiently at a low cost. One to two clinics are selected per state as sentinel sites to conduct both clinical-based and laboratory-based surveillance^4^. However, this traditional surveillance method used in Malaysia requires weeks or even months to gather, process, report, and finally release the surveillance data through WHO^5–7^. In addition, with only fifteen hospitals or clinics monitored^4^, the national ILI trend may not be captured accurately due to the small sample size and low coverage.

In recent years, there have been more research papers on influenza outbreak prediction. Many papers built prediction models based on historical ILI case data from traditional surveillance or WHO reports. These data had the limitations such as low geographic coverage and small sample size. Other papers used simulated data or Google Flu Trends (GFT) data. GFT was launched in 2008 to provide estimated influenza activities using Google searches^8^. GFT provided near real-time estimates of seasonal influenza activity each day and stimulated many innovative research projects.

In 2013, Dugas^9^ developed a model to forecast influenza cases number based on influenza data from one medical center. It was shown that the generalized linear autoregressive moving average (GARMA) model with Negative Binomial distribution integrating GFT information provided the highest influenza case forecast confidence at 83%. The model aims to provide advanced warning of future influenza cases for medical centers. However, it was tested on only one medical centre. Hence, its geographic generalizability must be further evaluated.

García^10^ used Bayesian model selection and Bayesian regression to detect outbreaks of ILI using surveillance data in 2015. Their method was applied to both Spanish influenza outbreaks in San Francisco, USA, in 1918 and acute respiratory illnesses (ARIs) from San Luis Potosí, Mexico, for validation. The paper claimed to have accurate and consistent predictions. However, the model performance evaluation was based on observations and lacked statistical measure reporting.

Bédubourg^11^ compared different statistical methods for early temporal detection of outbreaks by using R package surveillance on simulated data generated using a negative binomial model. Among all the models, the CUSUM generalized linear model (GLM) gave the best recall at 79.5% but had a very low precision value at 9.9%. Periodic Neg Binomial GLM gave the best precision value at 68.4% but had a very low recall value of 20.7%. All the tested models struggled to reach a high balanced score for both precision and recall. Therefore, they were either insensitive, missing out on real outbreaks, or overreacting to give many false alarms.

In 2019, Zhang^13^ combined GFT together with surveillance data FluNet published by WHO and developed a multivariate seasonal autoregressive integrated moving average model to track influenza epidemics in Australia, China, the USA, and the UK . In 2020, Darwish^12^ investigated the performance of three different feature spaces in different models to forecast the weekly ILI rate in Syria using Early Warning, Alert and Response System (EWARS) data from WHO. Both papers showed promising results. However, similar to traditional surveillance conducted by the government, the published WHO data could be delayed by several weeks or months.

In this paper, we propose a new data-driven methodology to detect and predict influenza outbreaks. A near real-time diagnostic dataset is used in this study, which covers information from over three thousand clinics in Malaysia. A new region index (RI) is developed to capture the ILI trend in the regions. By analysing the anomalies in the weekly RI value, potential outbreaks are identified using statistical methods. An ensemble learning method is developed to predict potential influenza outbreaks. Cross-validation is conducted to evaluate the prediction model performance. The proposed methodology is shown to be sensitive and accurate at influenza outbreak prediction, with around 80% accuracy, 75% recall, and 75% precision scores. It is also validated further with publicly available information, including the GFT data, news, and WHO FluNet data^6,7^.

## Methods

### Data

For this study, a dataset was provided by Zuellig Pharma, covering over 3000 clinics’ diagnostic records in their Malaysia clinic partner network. The dataset consists of approximately two million ILI case records from 4 Jan 2016 to 21 July 2019 with patient diagnostic details, such as the patient ID, diagnosis, prescription drugs, visiting date, etc. The pre-processing of diagnostic data is required to filter the relevant ILI data, because the raw dataset contains data from all types of illnesses. In this study, the ILI data were identified by ICD10 codes, the international classification of diseases codes used by the WHO^14^. To sound an early alarm on influenza outbreaks, both confirmed cases and early symptoms are considered. Table 1 shows the ICD10 codes used to filter the ILI data from the original diagnostic dataset.

**Table 1:** ICD10 Code and ILI Diagnosis.

ICD10	Diagnosis	Selected Reason
J09, J10, J11	Influenza	Patients diagnosed with influenza are included in the analysis to ensure specificity.
R50	Fever	According to Julia (2017)^15^, an ILI is defined by the WHO as “An acute respiratory illness with a measured temperature of > 38 $^{\circ }$C and cough, with onset within the past 10 days”, in which fever and cough are the two key diagnoses.
R05	Common Cough	
R06.7	Sneezing	Yang (2015)^16^ studied the key diagnosis associated with influenza. Fever + cough showed the best sensitivity and fever + cough + sneezing showed the best specificity at 77%. Therefore, sneezing was selected to increase specificity.
J00, J30	Common Cold	Charles (2016)^17^ noted that common cold and influenza normally share similar symptoms . Influenza patient might have been diagnosed as having the common cold at the beginning of their illness.

All the diagnostic data are collected automatically and refreshed in real time. The data can be aggregated into different frequencies, e.g., daily, weekly, or monthly. Similar to past surveillance systems, weekly data are used in this study. We aggregate the data by clinic and count the number of weekly ILI cases. So that, we can detect the weekly outbreak status and predict whether the next coming week will bring an outbreak. Our approach can provide outbreak alerts several weeks earlier than official reports from traditional surveillance methods.

### Region Index (RI)

**Figure 1:**
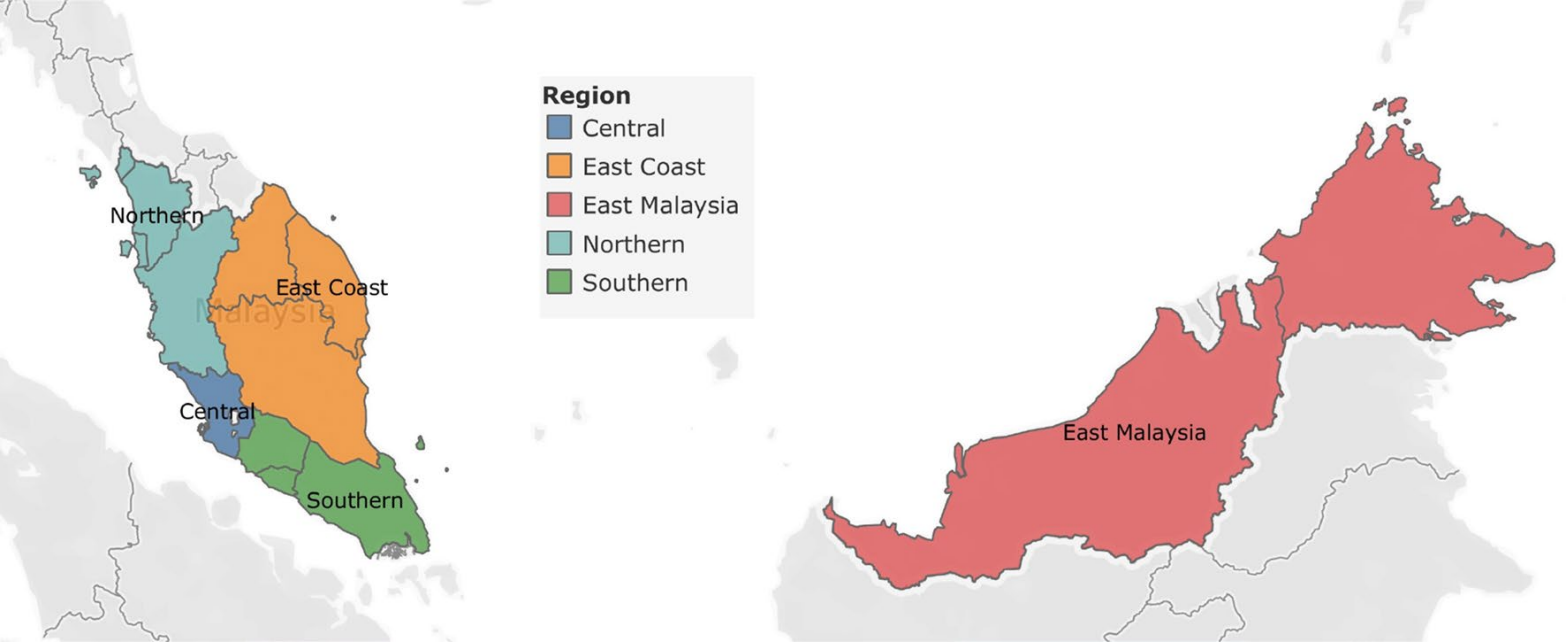
Five Regions in Malaysia (generated using Tableau version 2020.3.2) (https://www.tableau.com/support/releases/desktop/2020.3.2).

As the data is from the clinic partner network, it is common for new clinics to join the partner network from time to time. Therefore, the number of clinics in the dataset might be different over time. In addition, the clinics’ size could be varied from tens of patient visits a week to hundreds of patient visits a week. To study the regional influenza outbreak, we introduce the RI, a metric that normalizes the impact of the weekly number of clinics and the clinic size. Using the diagnostic data from the clinics, we have the flexibility to decide on the granularity of the regions by grouping the clinics based on geographic location. In this paper, we define five regions in Malaysia by following the definition used by the Malaysia Federal Department of Town and Country (Table 2)^18^. All clinics are segmented into five regions: the Central, East Coast, East Malaysia, Northern, and Southern regions (Fig. 1). After segmentation, each of the regions still has good coverage for the number of clinics and the number of ILI cases (Table 2). In a paper by Santillana^25^, the influenza surveillance for ten regions in the USA was studied and analysed independently. With the same concept, we will study each of the five regions independently in this paper.

**Table 2:** Definition of the Five Regions in Malaysia^18^.

Region	States	Number of clinics	Number of ILI cases
Central	Selangor	1,442	966,817
East Coast	Kelantan, Pahang, Terengganu	204	67,047
East Malaysia	Sabah, Sarawak	240	84,661
Northern	Kedah, Penang, Perak, Perlis	655	535,452
Southern	Johor, Melaka, Negeri Sembilan	506	350,115

The following data preparation step is to calculate RI for each of the five regions weekly. The mathematical definition of RI is shown in Equation (1). Table 3 shows an example of the regional RI.

1 $$\begin{aligned} R_{j,r}=\frac{\sum _{i=1}^{n_{j,r}} N_{i,j,r}}{\sum _{i=1}^{n_{j,r}} S_{i,j,r}} \text { , } j \ge 1 \end{aligned}$$

where

$R_{j,r}$ is Region Index (RI) at week *j* of region *r*

*r* $\in$ {Central, East Coast, East Malaysia, Northern, Southern} (Table 2)

$N_{i,j,r}$ is number of ILI cases of clinic *i* at week *j* of region *r*

$n_{j,r}$ is number of clinics at week *j* of region *r*

$S_{i,j,r}$ is average size of the clinic *i* at week *j* of region *r* as defined in Equation (2)

2 $$\begin{aligned} S_{i,j} = {\left\{ \begin{array}{ll} \frac{\sum _{J=k}^{J=j-1} N_{i,J}}{j-k} &{} \text {if } j > 1 ; (k \,\text { is the week number that clinic }\, i \,\text {joined the partnership)} \\ N_{i,1} &{} \text {if } j=1 \end{array}\right. } \end{aligned}$$

In Malaysia, no publicly available dataset contains all the clinics or hospitals. The data we use contain partner clinics, which may be of different sizes and may have joined the partnership at different times. By averaging the clinic sizes and normalizing based on this average number, we can avoid biases from missing data.

**Table 3:** Regional Index (RI) of Diagnostic Data.

Region	Date	Region Index
Central	Week 4 - 10 Jan 2016	1
Central	Week 11 - 17 Jan 2016	1.2
Central	.	.
Central	Week 15 - 21 July 2019	1.5
East Coast	Week 4 - 10 Jan 2016	1
.	.	.
Southern	Week 15 - 21 July 2019	1.6

### Influenza outbreaks detection method

**Figure 2:**
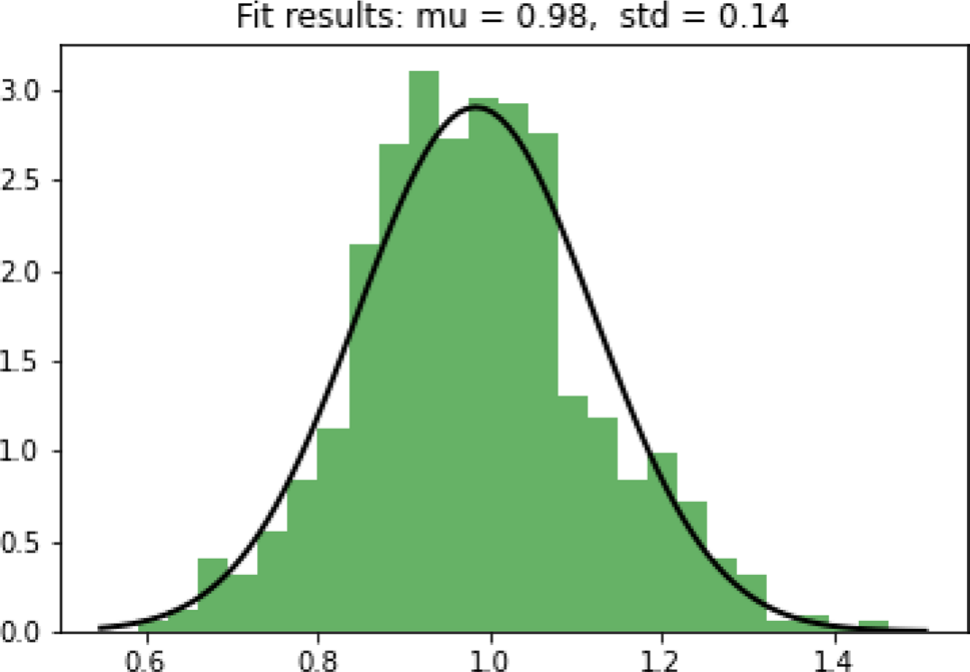
Histogram of RIs.

**Figure 3:**
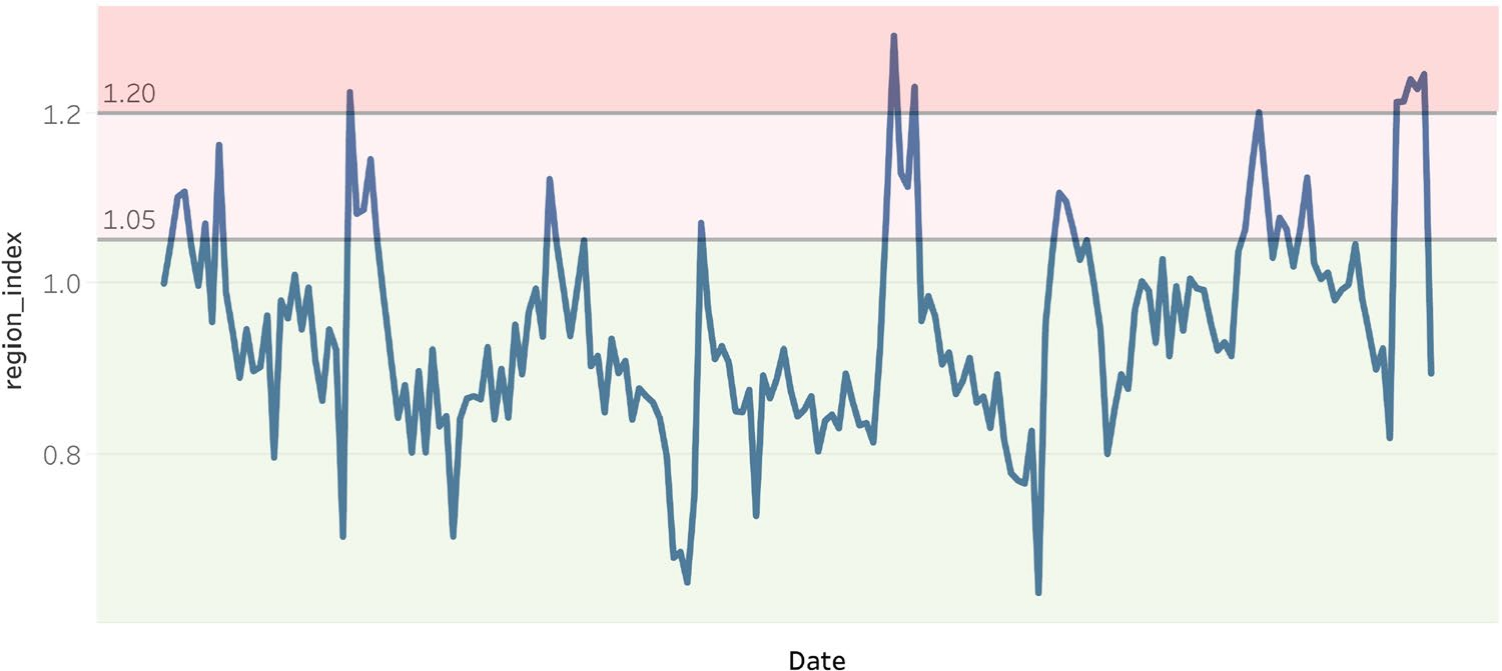
Example: Apply $70\%$ and $90\%$ threshold to Southern region’s RIs.

The RI had normalized the original ILI cases for each week and each region. A histogram is plotted below to show the distribution of RI, which is close to a normal distribution (Fig. 2). Next, we will apply anomaly detection models to label the regional outbreak on a weekly basis. From the past research papers^19–21^, the 70th and 90th percentiles are often used on normalized ILI cases to identify weak and strong indications of influenza outbreaks. Applying these thresholds to the data, $RI\ge 1.05$ and $RI \ge 1.2$ give weak and strong indications of influenza outbreaks at the 70th and 90th percentiles, respectively (Table 4). In the example illustration plot for the southern region, the weeks in the pink range represent strong indications of influenza outbreaks above the 90th percentile; the weeks in the light pink range represent weak indications of influenza outbreaks between the 70th and 90th percentiles (Fig. 3).

**Table 4:** Weekly RIs statistics Summary.

	Number of Weeks %				
RI Range	Central	East Coast	East Malaysia	Northern	Southern
RI $<1.05$	71%	71%	71%	61%	82%
RI in [1.05, 1.2)	23%	19%	21%	26%	13%
RI $\ge 1.2$	6%	10%	8%	14%	5%

These thresholds are used to design the anomaly detection models. The majority ($70\%$) of the RIs were below RI=1.05, representing a normal situation. A RI=1.05 will be used as the minimum requirement for a week to be labelled as an outbreak. (Equation (10)). A RI=1.2 is applied in one of the anomaly detection models. (Equation (3)).

#### Anomaly detection models

**Figure 4:**
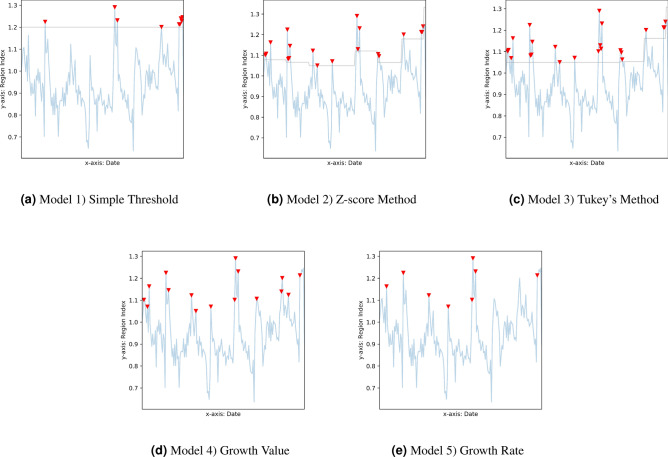
Outlier lables from five Statistics Models.

We consider influenza outbreaks as anomalies in the weekly RI values. To identify the anomalies, we use two types of statistical methods, including five statistical models (Table 5). The type A method is focused on detecting the anomalies over the statistical upper bound of the dataset, and the type B method identifies the abrupt growth in the time-series data. The type B method complements the type A method when the base values are low. The details of the anomaly detection models are described below. Here we use $O_{j,r,i}$ as the anomaly label at week *j* in the region *r* using model *i*. The southern region is used as an example, in which labelled weeks are plotted in red triangles for each of the five models (Fig. 4).

**Table 5:** Five Statistics Models used for Anomaly Detection.

Method	Explanation	Model	Feature
A. Outliers over Upper Bound	Statistical models that attempt to identify anomalies with value outside of the norm band	1) Simple Threshold: $RI >=1.2$	Interpretable and captures all extremely high RIs, i.e. RI over 90th percentile
		2) Z-score Model: $RI > 90\%$ Confidence Interval’s upper bound	Captures higher than upper bound points using the mean and standard deviation
		3) Tukey’s Model: $RI > 90\%$ IQR upper bound	Captures higher than upper bound points using quantiles
B. Abrupt Growth	Statistical models that attempt to identify anomalies that have abrupt growth	4) Growth Value: RI growth value > median (positive weekly growth value)	Captures abrupt growth in value
		5) Growth Rate: RI growth rate > 10%	Captures abrupt growth in percentage

- Model 1: Simple Threshold
  The first model is the simple threshold model. The anomaly label using the simple threshold model is given in Equation (3). RI=1.2 is selected as the threshold to give a 90% confidence interval (Table 4). This RI indicates that the labelled weeks have at least 20% more ILI cases than the historical average.
  3 $$\begin{aligned} O_{j,r,1} = {\left\{ \begin{array}{ll} 1 &{} \text {if } R_{j,r} \ge 1.2\\ 0 &{} \text {else} \end{array}\right. } \text { , } j \ge 1 \end{aligned}$$
- Model 2: Z-score Model
  In the Z-score model, the anomaly label is given in Equation (4). In each region, the $\mu$ and the $\sigma$ are the mean and the standard deviation of the RIs every half-year. p=1.3 is used for the 90th percentile (Equation (5)).

4 $$\begin{aligned} O_{j,r,2} = {\left\{ \begin{array}{ll} 1 &{} \text {if } R_{j,r} \ge \mu + p * \sigma \\ 0 &{} \text {else} \end{array}\right. } \text { , } j \ge 1 \end{aligned}$$

5 $$\begin{aligned} \Pr (O_{j,r,2} = 1) = 1 - Z_{score}(p) \end{aligned}$$

where:

$\mu$ is the mean of the RIs for each of the half-year (26 weeks) windows

$\sigma$ is the standard deviation of the RIs for each of the half-year (26 weeks) windows

*p* is a constant. In this paper $p=1.3$ is used to obtain a $90\%$ confidence interval using Equation (5)

- Model 3: Tukey’s Model
  The anomaly label using Tukey’s model is given in Equation (6). The confidence interval is computed using the quantiles of the RIs every half-year. Here, we use q=0.4 for the 90% confidence interval (Equation (7)).

6 $$\begin{aligned} O_{j,r,3} = {\left\{ \begin{array}{ll} 1 &{} \text {if } R_{j,r} \ge Q75 + q \times IQR \\ 0 &{} \text {else} \end{array}\right. } \text { , } j \ge 1 \end{aligned}$$

7 $$\begin{aligned} \Pr (O_{j,r,3} = 1) \xrightarrow [\text {}]{\text {normal approximation }} 1 - Z_{score}(0.6745 + q * 1.35) \end{aligned}$$

where *Q*75 is the 75th-Percentile of the RIs of the half-year (26 weeks) windows

*IQR* is the Z-score interquartile range of the RIs of the half-year (26 weeks) windows

*q* is a constant. In this paper $q=0.4$ is used to obatain a $90\%$ confidence interval using Equation (7)

- Model 4: Growth Value
  The fourth model uses the RI growth value as a measurement to identify the abruptly growing RIs. The anomaly label using the growth value is given in Equation (8). This values indicates that the labelled weeks have RI growth values exceeding the median of the positive growth values.

8 $$\begin{aligned} O_{j,r,4} = {\left\{ \begin{array}{ll} 1 &{} \text {if } R_{j,r} - R_{j-1,r} \ge median(R_{J,r} - R_{J-1,r}) \text { where } R_{J,r} > R_{J-1,r}\\ 0 &{} \text {else} \end{array}\right. } \end{aligned}$$

- Model 5: Growth Rate
  The fifth model uses the RI growth rate as a measurement to identify abruptly growing RIs. The anomaly label using the growth rate is given in Equation (9). This rate indicates that the labelled weeks have an RI growth rate exceeding 10%.

9 $$\begin{aligned} O_{j,r,5} = {\left\{ \begin{array}{ll} 1 &{} \text {if } \frac{R_{j,r} - R_{j-1,r}}{R_{j-1,r}} \ge 10\% \\ 0 &{} \text {else} \end{array}\right. } \text { , } j > 1 \end{aligned}$$

#### Outbreak labelling

**Figure 5:**
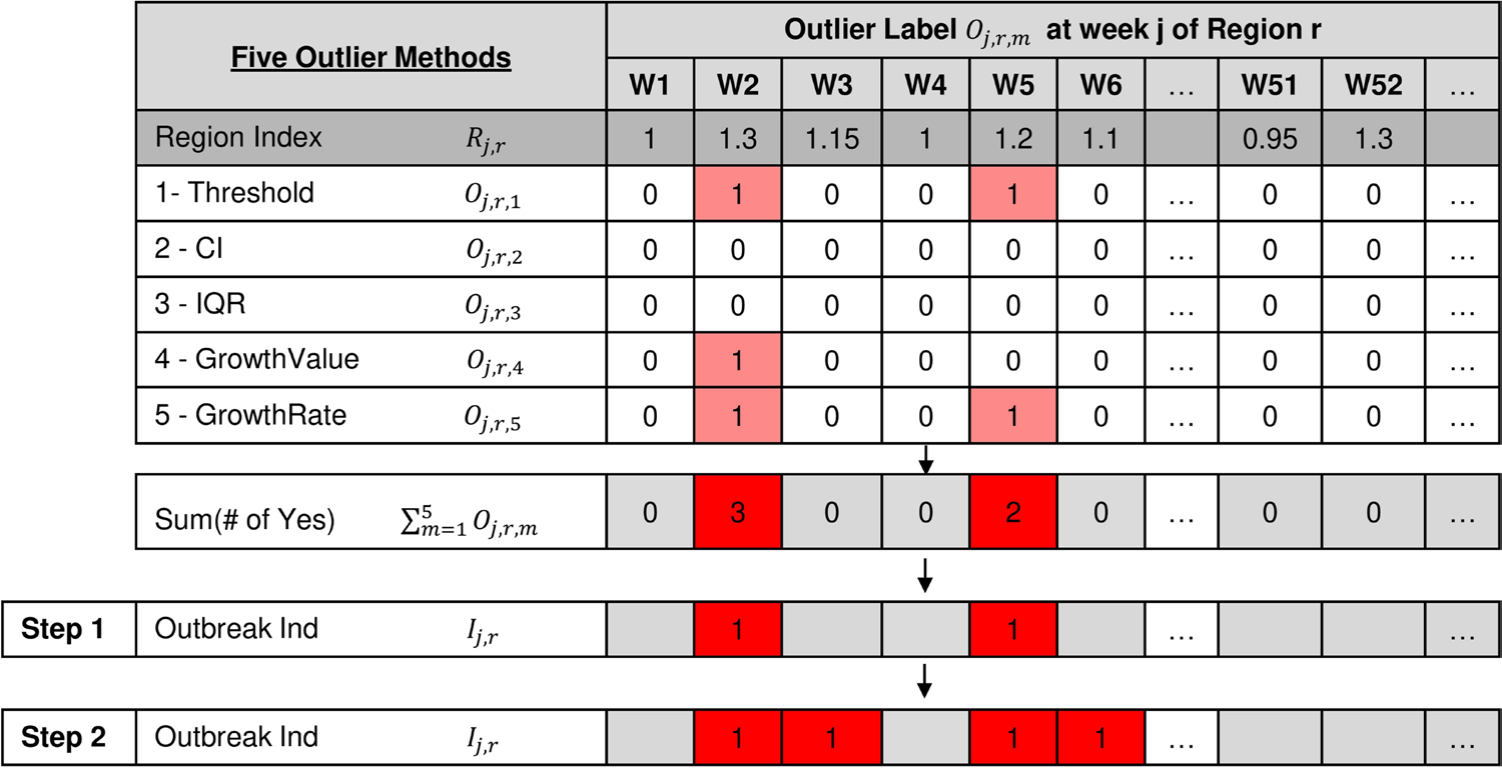
Influenza outbreak detection for historical data illustration.

The weekly outbreak labelling in each region is performed in two steps, as given by Equation (10) and (11) respectively. An illustration of this labelling process is shown in Fig. 5.

In the first step (Equation (10)), as long as one of the five anomaly detection models detects the anomaly, the week will be labelled as an outbreak. Because the five models complemented each other in anomaly detection, they increased the sensitivity of the influenza outbreak detection. Note that an $R_{j,r}$ greater than 1.05 from the 70*th* percentile is imposed, so that the weeks labelled as outbreaks were at least $5\%$ more frequent in ILI cases than the historical average to avoid over-labelling.

In the second step (Equation (11)), a two-week outbreak window is proposed in this paper. This is because the development of an infectious disease outbreak takes some time. Based on our study of the historical diagnostic dataset, for any region *r* that starts to show a strong indication of an outbreak in weeks $j-1$, the next week *j* will be considered as a continuity of the previous outbreak. In accordance with the empirical observation, this paper defines the start of an influenza outbreak as a two-week period.

10 $$\begin{aligned} I_{j,r} = {\left\{ \begin{array}{ll} 1 &{} \text {if } R_{j,r} \ge 1.05 \text { and } {\sum }_{m=1}^{m=5} O_{j,r,m} \ge 1 \\ 0 &{} \text {else} \end{array}\right. } \text { , } j \ge 1 \end{aligned}$$

where:

$I_{j,r}$ is the influenza outbreak indicator at week *j* of Region *r*,

1 means outbreak, and 0 means non-outbreak

11 $$\begin{aligned} I_{j,r} = {\left\{ \begin{array}{ll} 1 &{} \text {if } I_{j-1,r} =1\\ I_{j,r} &{} \text {else} \end{array}\right. } \end{aligned}$$

### Influenza outbreaks prediction method

With the labelled influenza outbreak data (Table 6), we develop an ensemble learning method^26^ to predict future outbreaks.

**Table 6:** Diagnostic data with labelled influenza outbreaks.

Region (*r*)	Date (*j*)	RI ($R_{j,r}$)	Influenza outbreaks ($I_{j,r}$)
Central	Week 4 - 10 Jan 2016	1	0
Central	Week 11 - 17 Jan 2016	1.2	1
Central	.	.	.
Central	Week 15 - 21 July 2019	1.5	1
East Coast	Week 4 - 10 Jan 2016	1	0
.	.	.	.
Southern	Week 15 - 21 July 2019	1.6	1

#### Feature generation—Focus on prior outbreak pattern

In supervised learning, feature X in response Y relationship must be constructed to train the model using historical data. Then, given a new X, the model can predict the corresponding Y. In this paper, response Y is the outbreak indicator of the next week. Feature X is constructed as the RI patterns of w weeks prior. Here, w is a parameter. This feature-response construction allows the model to address patterns before an outbreak.

Assuming there are n weeks of historical data available, Table 6 can be reformatted into Table 7 for each region r. The feature set X to respond to Y construction is shown in Equation (12) for historical data. In each region r, X takes the past w weeks’ RI, and Y is the influenza outbreak indicator. The same construction works to predict future outbreaks, as shown in Equation (13). Given the known $X_{n-w+1}$, i.e., the most recent *w* weeks’ RI from the historical data, the classification model predicts unknown $Y_{n-w+1}$, i.e., the outbreak indicator of the next week.

Note that the (X, Y) pairs are mutually independent. It has been assumed that the outbreak indicator of week j only depends on the RI pattern of the week j-w to week j-1. In other words, it is the week j-w to week j-1’s RI pattern that decides whether week j is an outbreak. That is why parameter w must be selected with care. We will discuss the use of cross-validation to select the optimal value for parameter w.

**Table 7:** Reformat of Table 6 for Each Region r.

Date Week (*j*)	$W_1$	$W_2$	$W_3$	.	$W_{n-1}$	$W_n$
RI ($R_{j,r}$)	$R_1$	$R_2$	$R_3$	.	$R_{n-1}$	$R_n$
Outbreak Indicator ($I_{j,r}$)	$I_1$	$I_2$	$I_3$	.	$I_{n-1}$	$I_n$

12 $$\begin{aligned} \begin{aligned} (X_u, Y_u) = ((R_u, R_{u+1},..., R_{w+u-1}) , (I_{w+u})) \text { for } 1 \le u \le n-w \end{aligned} \end{aligned}$$

where:

$X_u$ is the feature constructed at week $w+u$, which is composed of RIs from *w* weeks prior

$Y_u$ is the response at week $w+u$, which is the outbreak indicator

13 $$\begin{aligned} (X_{n-w+1}, Y_{n-w+1}) = ((R_{n-w+1}, R_{n-w+2},..., R_{n}) , (I_{n+1})) \end{aligned}$$

#### Model design

Response Y, the outbreak indicator, is a 1/0 binary variable, where 1 represents outbreaks and 0 represents non-outbreaks. This paper uses an ensemble learning method with a support vector machine (SVM) and Gaussian naive Bayes (GNB) and a simple OR function as the combiner^26^. The pseudocode of the ensemble learning method is shown in Algorithm 1.

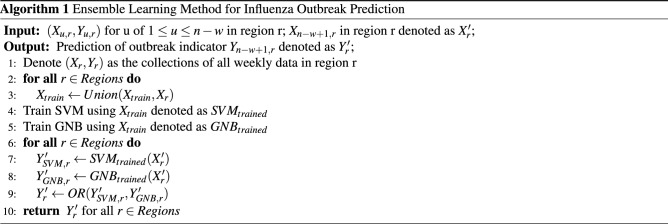



SVM is selected because it fits perfectly into the paper’s problem setup. As it is widely known, SVM can be used in supervised learning, which plots each example X as points in space. Its aim is to find a hyperplane to separate the points by category Y as widely as possible. With the hyperplane, new example X entering the space will fall to one side of the hyperplane, therefore being predicted to belong to a category Y. The same concept is then applied to the data structure defined in section ”Feature Generation - Focus on Prior Outbreak Pattern”. For features constructed using historical data as described in Equation (12), each example X is a point in space. SVM aims to find a hyperplane to separate Y=1 outbreaks from Y=0 non-outbreaks as widely as possible. With the hyperplane, the Equation (13) new X (X is the last W weeks RI) entering the space will be categorized to be either Y=1 or Y=0 (Y is the prediction for next week’s outbreak indicator).

SVM might fail to separate outbreak cases from non-outbreak cases if the pattern for Y is not so distinct. If relying purely on SVM, it might lead to false negative predictions that the model may fail to capture all outbreaks correctly. In this paper, we select one more classification model to complement the SVM model, GNB. GNB can be applied because we have shown that the RI value follows a Gaussian distribution (Figure 2). It uses Bayes theorem for prediction using conditional probability function and is able to capture outbreaks that SVM might miss out from a different angle.

#### Model training and parameter tuning

**Figure 6:**
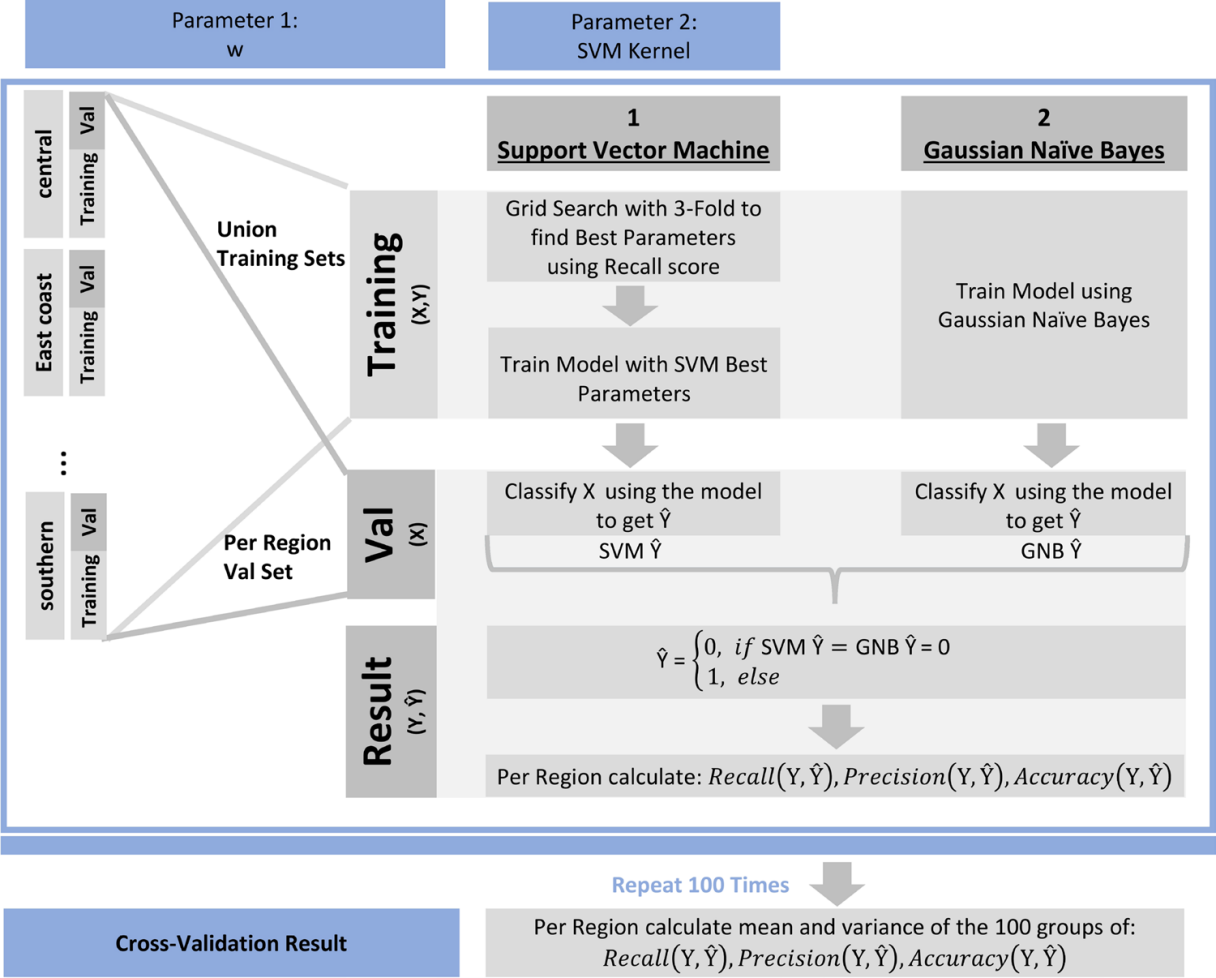
Cross-validation architecture.

To build a robust prediction model and provide an unbiased performance evaluation of the final model, we divide the whole data set into training, validation, and test data sets. 15% of the data are set aside as a test dataset. The remaining 85% of the data are used in a cross-validation process, which is further split into training (70%) and validation (30%) data. Details about the cross-validation process using repeated random sub-sampling^27^ are described in Algorithm 2 (Fig. 6) to optimize the hyperparameter *w* and SVM *kernel*.

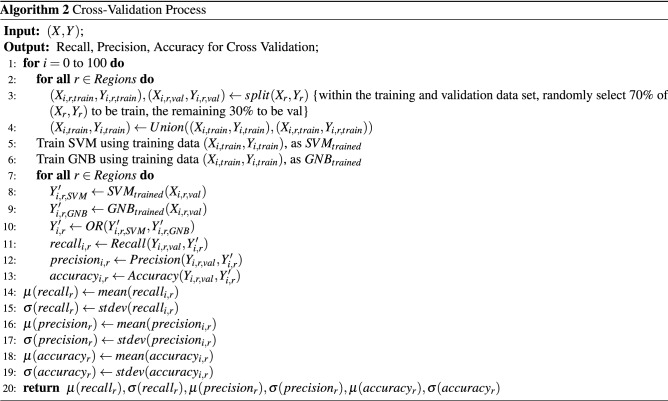



Three evaluation metrics are selected to be reported for model performance evaluation in this paper.

1. Recall score as the primary metrics aiming to find all real outbreaks
  The recall score measures how sensitive the model is at reporting actual outbreaks, i.e., how many real outbreaks are being predicted correctly by the model. The primary purpose of the project is to detect potential outbreaks early and not miss any actual outbreaks. The historical data are imbalanced in the outbreak indicator labelling, and there are many more 0s (non-outbreak weeks) than 1s (outbreak weeks). Therefore, the recall score is the optimal option for evaluating the sensitivity of the model in identifying real outbreaks. The recall score is used in the cross-validation process for parameter tuning and to increase the model sensitivity.
2. Precision score to ensure predicted outbreaks are real outbreaks
  The precision score measures the precision of the model in reporting outbreaks, i.e., for all the weeks predicted as an outbreak by the model, how many are real outbreaks. The precision score is reported together with the recall score during cross-validation to avoid over-labelling of the outbreaks. It is also reported as the confidence level of the prediction result, indicating the probability of the predicted outbreak being a real outbreak.
3. Accuracy score for reference
  Accuracy is the most intuitive performance measure. Due to the nature of imbalanced data, the accuracy is quite high in general. Therefore, it is reported just for reference.

We use Algorithm 2 to select the best hyperparameters for the prediction model. The cross-validation result of the recall score is shown in Table 8. SVM $kernel=rbf$ and $w=3$ are selected as the best parameters, because they provide high, balanced, and stable recall scores across all five regions. Table 9 reports the precision and accuracy scores on top of the recall scores for the selected best hyperparameters. For all the regions, there are high recall and precision scores and low standard deviations. Once we obtain the optimal hyperparameter set, we use the test data set to measure the final model performance, which is further elaborated in the results section.

**Table 8:** Cross-validation result of recall score.

Parameters		Recall score: means (standard deviation)				
Kernel	w	Central	East Coast	East Malaysia	Northern	Southern
poly	2	0.56 (0.12)	0.68 (0.10)	0.68 (0.11)	0.69 (0.10)	0.60 (0.16)
poly	3	0.58 (0.13)	0.75 (0.10)	0.73 (0.10)	0.69 (0.01)	0.71 (0.13)
poly	4	0.58 (0.11)	0.75 (0.11)	0.72 (0.11)	0.67 (0.10)	0.65 (0.13)
poly	5	0.58 (0.13)	0.76 (0.12)	0.68 (0.13)	0.61 (0.09)	0.60 (0.16)
poly	6	0.60 (0.13)	0.71 (0.10)	0.77 (0.12)	0.64 (0.09)	0.63 (0.14)
rbf	2	0.67 (0.11)	0.74 (0.09)	0.71 (0.11)	0.75 (0.08)	0.66 (0.15)
rbf	3	0.65 (0.14)	0.74 (0.10)	0.75 (0.11)	0.74 (0.11)	0.71 (0.14)
rbf	4	0.67 (0.12)	0.75 (0.10)	0.72 (0.11)	0.74 (0.10)	0.68 (0.13)
rbf	5	0.67 (0.12)	0.82 (0.11)	0.74 (0.12)	0.70 (0.10)	0.62 (0.16)
rbf	6	0.66 (0.11)	0.77 (0.10)	0.77 (0.12)	0.69 (0.10)	0.64 (0.14)

**Table 9:** Detailed Cross-Validation results with optimal parameter: Kernel = rbf, w=3.

	Central	East Coast	East Malaysia	Northern	Southern
Recall	0.65 (0.14)	0.74 (0.10)	0.75 (0.11)	0.74 (0.11)	0.71 (0.14)
Precision	0.74 (0.10)	0.63 (0.11)	0.72 (0.12)	0.72 (0.10)	0.73 (0.12)
Accuracy	0.81 (0.04)	0.80 (0.05)	0.83 (0.05)	0.78 (0.06)	0.89 (0.04)

### Ethical use of data

The authors confirm that all methods were carried out in accordance with relevant guidelines and regulations. All the experimental protocols were approved by Zuellig Pharma Holdings Pte Ltd and National University of Singapore. Written informed consent was obtained from all subjects by the approving ethics committee. The consent of use of the data for this study by the authors has been provided by Zuellig Pharma Holdings Pte Ltd.

## Results

We compared the model outbreak detection and prediction results with both the WHO and the GFT data. Currently, Malaysia Ministry of Health (MOH) reports ILI cases to WHO based on the traditional surveillance method^4^. A total of 15 clinics or hospitals in the whole country monitor and report ILI cases to WHO every two weeks or longer. The WHO published weekly influenza data including the total number of specimens processed and the total number of confirmed influenza cases on FluNet^7^. In this study, we used WHO data to validate the high-level ILI trend at the national level.

Although the WHO data is able to show some useful national ILI trends, it is very approximate due to the small sample size. In addition, the WHO data cannot support the detailed weekly analysis at the regional level. Therefore, we compared the regional detection and prediction results with the GFT data. As an example of collective intelligence, GFT has attracted a lot of attention in the past 10 years^28,29^. On the one hand, GFT unlocked the power of big data in the public health area. It is able to understand the prevalence of influenza at very local levels with more finely granular data from search engines, which is not practical for the traditional surveillance systems to widely produce. In addition, GFT can provide influenza alerts much earlier than the traditional surveillance systems. On the other hand, GFT was challenged by some researchers because its predictions have sometimes been inaccurate. This is because that people making influenza-related Google searches may know very little about how to diagnose influenza. Therefore, searches for influenza or influenza symptoms may well be researching disease symptoms that are similar to influenza but are not influenza.

The GFT data are selected in this study for comparison for the following reasons. Firstly, GFT is one of the best public data sources for providing near real-time influenza information at very local levels. GFT can specify search terms and geographic granularity to align with our model at the regional level. We can use ILI-relevant search terms and choose the cities in the same region from GFT to provide the closest comparison with our model results. Secondly, despite its limitations, GFT is still able to provide useful influenza alert information in many cases based on previous research^8,28,29^. Thirdly, the potential problem of GFT can be identified and rectified as we also use the WHO data to validate the national-level results in this study.

### Result of the Influenza outbreaks detection method

**Figure 7:**
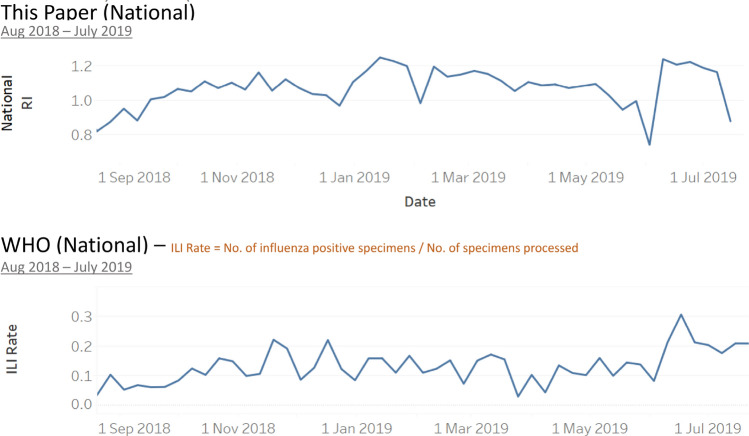
National level RI vs. WHO weekly influenza rate in the past 1 year.

We extracted Malaysia’s influenza data from WHO FluNet website^7^ and computed the influenza rate as the percentage of influenza cases found in all the processed specimens. We calculated the aggregated RI at the national level and compared it with WHO influenza rate data trend. Overall, these national-wide data are aligned and show a similar trend (Fig. 7).

**Figure 8:**
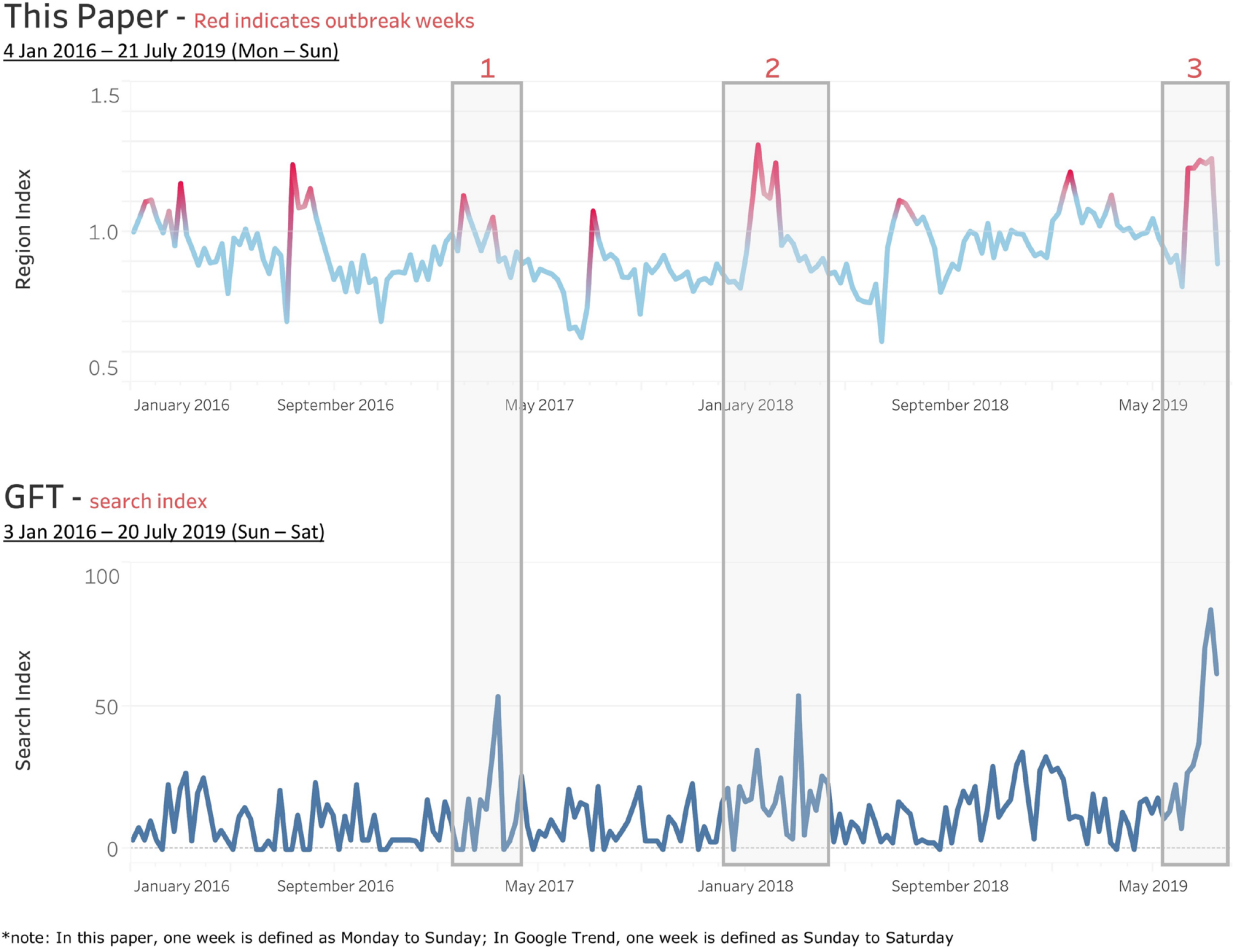
Outbreak detection result vs GFT for the southern region.

To validate the influenza detection results at the regional levels, GFT data are used. Figure 8 shows an example of the comparison in the southern region. As defined in Table 2, the region is composed of three states: Johor, Melaka, and Negeri Sembilan. The upper graph shows the influenza outbreaks in the southern region as labelled using the approach proposed in this paper (red color indicates outbreaks). The lower graph shows the GFT search index of ILI-relevant terms for the same region. From this comparison, the GFT shows outbreak periods similar to those of our model. However, as we used real ILI case data from more than three thousand clinics while GFT relied on the search results from the general public, our method was able to detect the outbreaks more obviously in most cases while GFT patterns were not so clear in some cases.

In Fig. 8, we highlighted three local outbreaks in the southern region, in which GFT also shows distinct spikes in the search index. To further validate the outbreak detection results, we also collected published news reports. In week 27 of 2019 (from 30 June to 6 July), the Selangor Journal reported that ILI cases soared in Negri Sembilan^22^, which is a state in the southern region. The finding is aligned with the model detection result, as specified in Fig. 8 outbreak period 3. In addition, we compared the time at which our paper gives the signal with that of the GFT for the 3 highlighted outbreaks, as summarized in Table 10. From the comparison, our method could detect the same outbreak during the same week or earlier than the GFT.

**Table 10:** First spike date comparison of this paper’s outbreak detection vs. the GFT for the southern region in sample periods.

Outbreak ID	Our Paper	GFT	Conclusion
1	Week 30 Jan 2017	Week 12 March 2017	Our paper detects outbreaks earlier than the GFT
2	Week 15 Jan 2018	Week 14 Jan 2018	Our paper detects outbreaks at the same week as the GFT
3	Week 10 Jun 2019	Week 7 July 2019	Our paper detects outbreaks earlier than the GFT

### Result of the Influenza outbreaks prediction method

To provide an unbiased evaluation of the prediction model, we use a testing data set to measure the performance of the model trained early on. The testing data set includes 15% of the total data, which are not used in the model training and validation stage. Model performance results (Table 11) are summarized below.

- The model is proven to be reliable and stable, with average of 75% recall, 74% precision and 83% accuracy scores across the five regions. (Table 11).
- The model is sensitive to capturing the real outbreaks, that around 75% of the real outbreaks can be correctly predicted by the model across the five regions (Table 11. Recall).

**Table 11:** Testing Data Set Results with Optimal Parameter: Kernel = rbf, w=3.

	Central	East Coast	East Malaysia	Northern	Southern
Recall	0.78	0.86	0.80	0.67	0.63
Precision	0.88	0.60	0.89	0.60	0.71
Accuracy	0.90	0.83	0.90	0.67	0.83

To further validate the prediction model performance, we predict whether the next week, i.e., week 22 to 28 Jul 2019, would be an outbreak for each of the five regions by using the historical data from 4 Jan 2016 to 21 Jul 2019. The model predicts that East Malaysia would not have an influenza outbreak in the next week, and all the rest of the regions will have an influenza outbreak then (Table 12). To be specific, there is around 88% of probability central will have an outbreak; 60% of probability east coast will have an outbreak; 89% of probability east Malaysia will have an outbreak; 60% of probability northern will be an outbreak; and 71% of probability southern region will have an outbreak (Table 11).

**Table 12:** Predicted Influenza Outbreaks for Next Week 22 to 28 Jul 2019 with Optimal Parameter: Kernel = rbf, w=3 1 represents outbreak, and 0 represents non-outbreak.

	Central	East Coast	East Malaysia	Northern	Southern
Predicted	1	1	0	1	1

**Figure 9:**
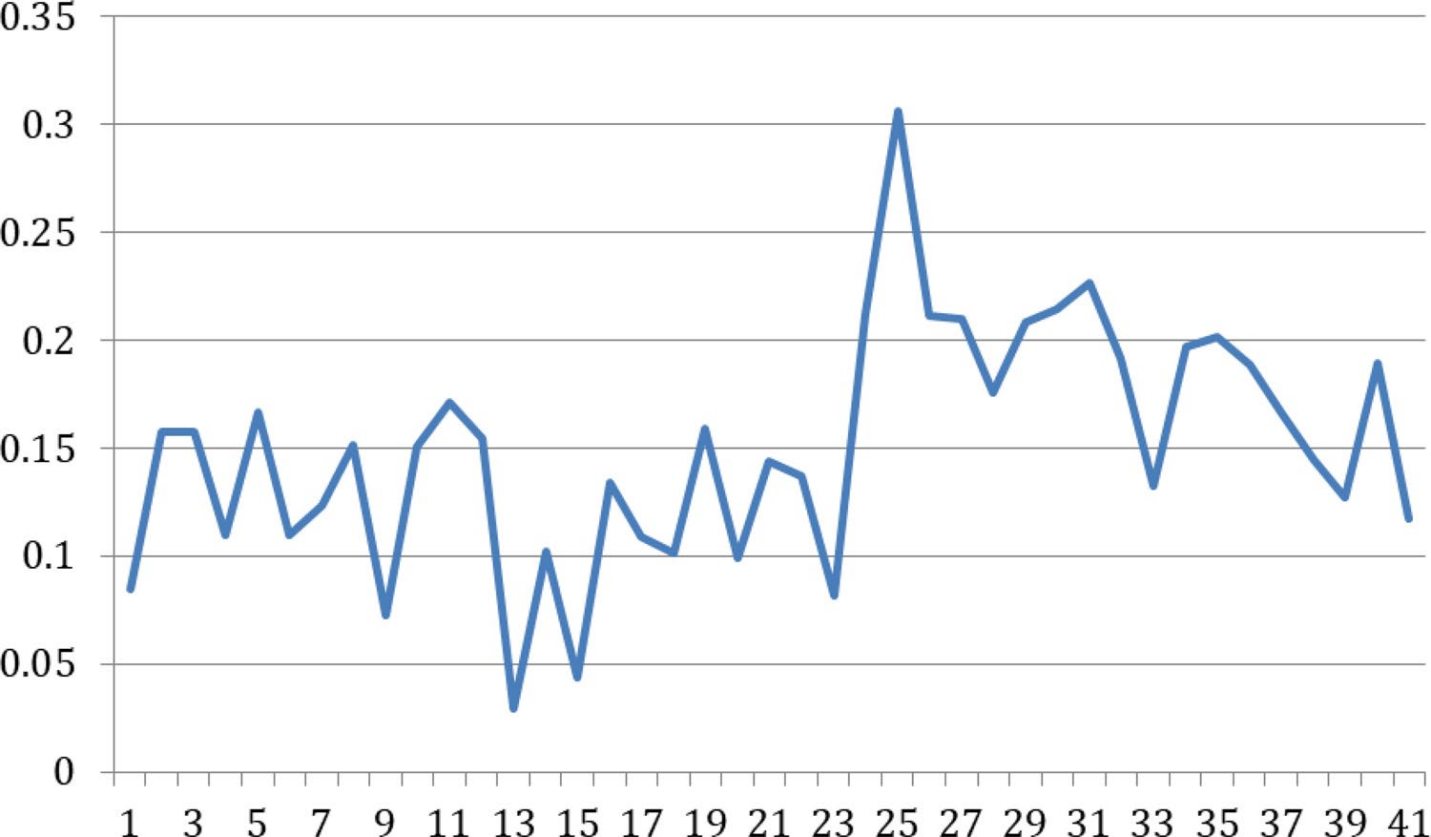
WHO weekly influenza rate in 2019.

We also compared the new prediction result with WHO FluMart data in the national level (Fig. 9). It shows that there is a spike around week 30 (22 to 28 Jul 2019), which is consistent with our prediction.

**Figure 10:**
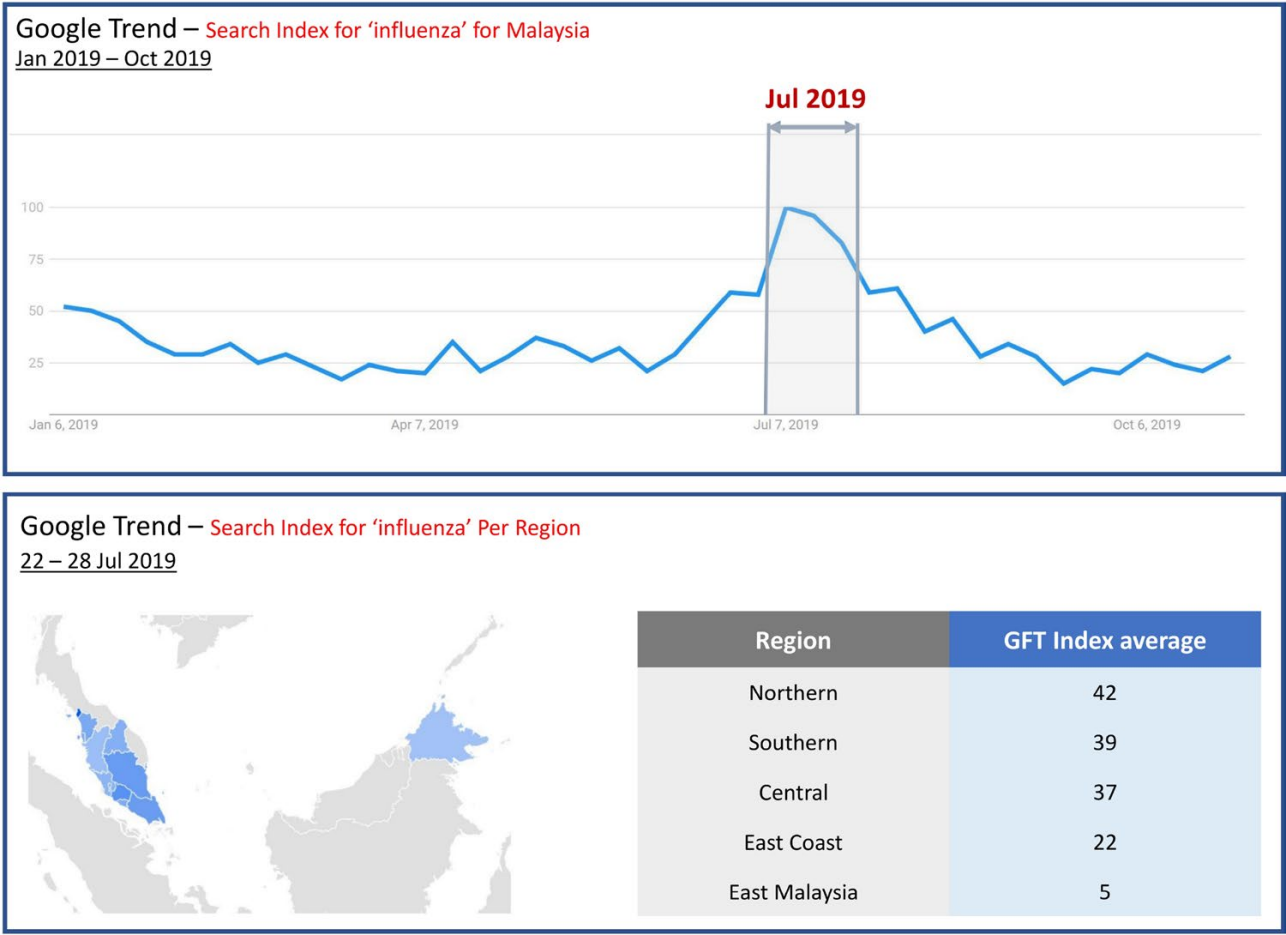
Google Flu Trend Result (week 22 – 28 Jul 2019) (Map screenshot from GFT website^23^).

From the GFT data (Fig. 10), we can see that there is also a distinct spike in searches for ’Influenza’ for Malaysia overall in the week of 22 to 28 Jul 2019. This is consistent with the prediction result from our model (Table 12). More specifically at the region level:

- Northern, southern, central and east coast regions have the top GFT Indexes indicating high influenza searches, which is consistent with the model result that these regions will have an outbreak (Table 12).
- East Malaysia has the lowest GFT index with the least likelihood of an outbreak, which is consistent with the model result showing 0, no outbreak (Table 12).

## Discussion

This paper proposes a data-driven methodology using a diagnostic dataset from over 3000 clinics in Malaysia to detect and predict influenza outbreaks at very local levels effectively. The main objective of this study is to provide a good supplement to traditional influenza surveillance systems instead of a replacement. We believe that when our method is deployed in conjunction with traditional surveillance systems and GFT, it will provide better influenza detection and prediction outcomes.

**Figure 11:**
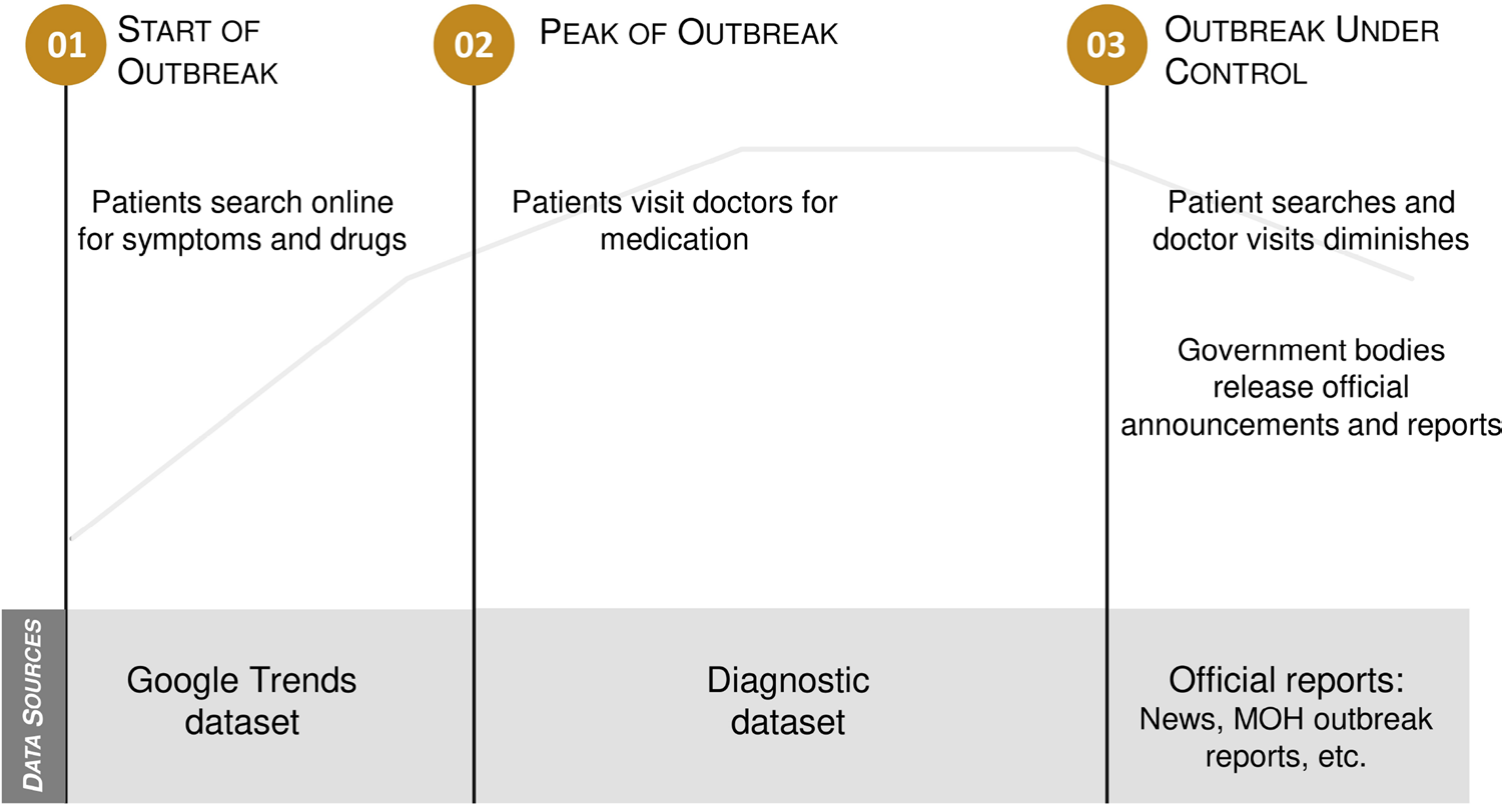
A typical outbreak lifecycle.

During a typical outbreak life-cycle as plotted in Fig. 11, there are three types of datasets that can be used for the influenza outbreak study: Google Trends dataset, diagnostic datasets, and official reports. Diagnostic data stand out for the following reasons. First, they are used to monitor ILI cases based on licensed doctor diagnoses, which are usually more reliable than Google Trends search-based data. Second, diagnostic data provide earlier detection insights into influenza outbreaks compared to the official reports. Andrea validated that diagnostic data from one medical center work well for the influenza case forecasting^9^. In this study, we used diagnostic data from over three thousand clinics, covering half of the clinics in Malaysia^24^. We can extend Andrea’s work to address geographic generalizability. In addition, as this dataset is directly extracted from a real-world healthcare system, our methodology can be easily integrated with this system and deployed for daily operations.

A new measurement metric of influenza outbreaks, i.e., the RI, is proposed in this paper. The RI compares the number of ILI cases during the current week with the historical weekly average in the region. Unlike previous papers^13,19,21^, which simply use the total number of cases from all hospitals or clinics, the RI uses the average size of individual clinics and handles cases when new clinics are added into or removed from the dataset during the sample period. In statistical terms, the RI eliminates the biases of different clinic sizes and a varying number of clinics. It gives a good indication of whether the current week shows an anomaly in the ILI cases in the region.

We designed an influenza outbreak detection method based on RI using statistical outlier detection models and validated the method with GFT results. In real-world scenarios, the sensitivity of the outbreak detection model is crucial because we try to detect as many outbreaks as possible. To improve the sensitivity of outbreak detection methods, we introduce five complementary statistical models in this paper. These models are used to label the weekly regional outbreaks to train the prediction model.

This paper emphasizes the study of RI patterns before an outbreak and develops a machine learning model to predict future outbreaks. There are usually two types of methodologies when detecting outbreaks, i.e., regression models and classification models. Regression models focus on seasonal or periodical outbreaks and fit long-term predictions better. By contrast, classification models capture dynamic patterns and fit short-term predictions better. In this paper, we decided to use classification models because Malaysia, which is located in Southeast Asia, does not have distinctive seasons; therefore, there are no clear seasonal trends in ILI cases^4^. Moreover, we can give early alerts using classification models by learning the patterns right before the outbreaks.

The methodology has consistent performances, with average of $75\%$ recall, $74\%$ precision, and $80\%$ accuracy scores on the testing set across five regions in Malaysia. Therefore we conclude that the methodology is sensitive and accurate in predicting influenza outbreaks at very local levels. Compared to previous similar research works, the proposed methodology is more reliable, effective, and scalable to influenza outbreak detection and prediction.

In the future, the proposed methodology introduced in this paper can be easily adapted to other diseases covered by diagnostic data, such as hand foot mouth disease, dengue fever, and COVID-19, etc. Moreover, the methodology, as currently predicting at the regional level, can be extended easily to the city level, or any granularity by grouping clinics based on geographic location. In addition to the short-term prediction results presented in this paper, the methodology can be extended for long-term outbreak prediction by restructuring the data and incorporating other machine learning models. In addition, we can apply more complex nonlinear models such as LSTM or other neural networks to the data set to explore if they have better prediction accuracy.

## References

[1] World Health Organization. *Ten Health Issues WHO Will Tackle This Year*, https://www.who.int/news-room/feature-stories/ten-threats-to-global-health-in-2019 (2019).

[2] World Health Organization. *Influenza (Seasonal)*, https://www.who.int/en/news-room/fact-sheets/detail/influenza-(seasonal) (2018).

[3] Ong MP, Sam IC, Azwa H, Mohd Zakaria IE, Kamarulzaman A, Wong MH, Syed Omar SF, Hussain SH (2010). High direct healthcare costs of patients hospitalised with pandemic (H1N1) 2009 influenza in Malaysia. J. Infect..

[4] Sengol Selvanesan, Norizah Ismail, Yusof Mohd Apandi, Thayan R. MALAYSIA INFLUENZA SURVEILLANCE PROTOCOL. https://www.researchgate.net/publication/329023936_MALAYSIA_INFLUENZA_SURVEILLANCE_PROTOCOL (2018).

[5] World Health Organization. *Influenza update*, https://www.who.int/influenza/surveillance_monitoring/updates/latest_update_GIP_surveillance/en (2020).

[6] World Health Organization. *FluNet Summary*, https://www.who.int/influenza/gisrs_laboratory/updates/summaryreport/en/ (2020).

[7] WHO FluMart platform, https://apps.who.int/flumart/Default?ReportNo=12

[8] Ginsberg J, Mohebbi M, Patel R (2009). Detecting influenza epidemics using search engine query data. Nature.

[9] Andrea Dugas, Mehdi Jalalpour, Yulia Gel, Scott Levin, Fred Torcaso, Tak Igusa, Richard Rothman (2013). Influenza Forecasting with Google Flu Trends. PLoS ONE.

[10] García YE, Christen JA, Capistrán MA (2015). A bayesian outbreak detection method for influenza-like illness. BioMed Res. Int..

[11] Bédubourg G, Le Strat Y (2017). Evaluation and comparison of statistical methods for early temporal detection of outbreaks: A simulation-based study. PLoS ONE.

[12] Darwish A, Rahhal Y, Jafar A (2020). A comparative study on predicting influenza outbreaks using different feature spaces: application of influenza-like illness data from Early Warning Alert and Response System in Syria. BMC Res. Notes.

[13] Zhang Y, Yakob L, Bonsall MB (2019). Predicting seasonal influenza epidemics using cross-hemisphere influenza surveillance data and local internet query data. Sci. Rep..

[14] World Health Organization. *ICD10 code for Diagnosis*, https://icd.who.int/browse10/2016/en (2016).

[15] Fitzner J, Qasmieh S, Mounts AW, Alexander B, Besselaar T, Briand S, Brown C, Clark S, Dueger E, Gross D, Hauge S, Hirve S, Jorgensen P, Katz MA, Mafi A, Malik M, McCarron M, Meerhoff T, Mori Y, Mott J, Vandemaele K (2018). Revision of clinical case definitions: influenza-like illness and severe acute respiratory infection. Bull. World Health Organ..

[16] Yang JH, Huang PY, Shie SS, Yang S, Tsao KC, Wu TL, Leu HS, Huang CT (2015). Predictive Symptoms and Signs of Laboratory-confirmed Influenza: A Prospective Surveillance Study of Two Metropolitan Areas in Taiwan. Medicine.

[17] Charles Patrick Davis, *Cold vs. Flu*, https://www.medicinenet.com/cold_vs_flu/article.htm#cold_vs_flu_facts (2016).

[18] MLIT, *An Overview of Spatial Policy in Asian and European Countries - Malaysia*. https://www.mlit.go.jp/kokudokeikaku/international/spw/general/malaysia/index_e.html (2015).

[19] Pung R, Lee VJM (2020). Implementing the World Health Organization Pandemic Influenza Severity Assessment framework–Singapore’s experience. Influenza Other Respirat. Viruses.

[20] ElGawad BA, Vega T, El Houssinie M, Mohsen A, Fahim M, ElSood HA, Jabbour J, Eid A, Refaey S (2020). Evaluating tools to define influenza baseline and threshold values using surveillance data, Egypt, season 2016/17. J. Infect. Public Health.

[21] Guo P (2017). Monitoring seasonal influenza epidemics by using internet search data with an ensemble penalized regression model. Sci. Rep..

[22] 15 Clusters of ILI Detected in N. Sembilan as of 6 July. *Selangor J.*. https://selangorjournal.my/2019/07/15-clusters-of-ili-detected-in-n-sembilan-as-of-6-july/ (2019).

[23] Google Trend data – search term ‘influenza’. https://trends.google.com/trends/explore?q=influenza

[24] Makmor T, Khaled T, Ahmad Farid O, Nurul Huda MS (2018). Demographic and socioeconomic factors associated with access to public clinics. J. Health Transl. Med..

[25] Santillana M, Nguyen A, Louie T (2016). Cloud-based Electronic Health Records for Real-time. Region-specific Influenza Surveillance. Sci. Rep..

[26] Kuncheva LI. Classifier ensembles for changing environments. In International Workshop on Multiple Classifier Systems, LNCS 3007. Springer, (2004).

[27] Picard R, Cook R (1984). Cross-validation of regression models. J. Am. Stat. Assoc..

[28] Kandula S, Shaman J (2019). Reappraising the utility of Google Flu Trends. PLoS Comput. Biol..

[29] Olson DR, Konty KJ, Paladini M, Viboud C, Simonsen L (2013). Reassessing Google Flu Trends Data for Detection of Seasonal and Pandemic Influenza: A Comparative Epidemiological Study at Three Geographic Scales. PLoS Comput. Biol..

